# A Case of Iodic Salivation

**Published:** 1882-07

**Authors:** W. S. Elkin

**Affiliations:** Atlanta, Ga.


ATLANTA
MEDICAL REGISTER.

VOL. I.] JULY, 1882. [No. 10

### Original.

### A CASE OF IODIC SALIVATION.

BY W. S. ELKIN, M.D., ATLANTA, GA.

This affection cannot be very frequent, for many practitioners have used iodine in its several forms for years without ever having witnessed it. But since I have had the good fortune to see a typical case of salivation from iodide of potassium, I am persuaded to report the case.

Mr. C——, aged twenty-four years, tall and thin, of marked nervous temperament, and in the enjoyment of good health until six years ago, when he was attacked with syphilis. As soon as the eruptions characteristic of the secondary stage appeared, and a correct diagnosis of the disease was made, he was put upon a preparation of the protiodide of mercury, which treatment was kept up at irregular intervals, however, for nearly two years. At the expiration of this time the patient visited the Hot Springs, where he remained about four months, and then returned home apparently a well man.

On the evening of the 28th of April, of this year, he came to my office complaining of a dull aching pain in the anterior portion of the lower third of the tibia. Examination showed a slight circumscribed swelling, accompanied by pain, heat, and tenderness on pressure. The pain was more intense at night. It was easily discovered that he was suffering from a case of periostitis, which is a frequent consequence of syphilis. I applied counter-irritation in the form of tincture of iodine to the affected part, and ordered ten grains of iodide of potassium to be taken three times a day after eating, with instructions that at the end of two weeks the dose should be increased to a teaspoonful and a half of the solution, or fifteen grains, three times a day. As he was anæmic, I also prescribed a tonic preparation of iron and cod liver oil.

On the evening of the second day after this, the patient came to my office suffering with headache and intense pain in the mouth and region of the throat. Upon inquiry I discovered that instead of taking as directed, a teaspoonful of the solution which contained ten grains of iodide of potassium, he had suddenly increased the amount to a teaspoonful and a half, and thus received fifteen grains at a dose. This was taken about an hour after dinner. Two hours after the ingestion of this dose the patient began sneezing, which lasted for some fifteen or twenty minutes. He was then seized with violent pains in the roof of his mouth, accompanied by intense aching and seeming loosening of all the teeth. In moving the head from side to side excruciating pain was produced along both sterno-cliedo-mastoid muscles, and in the muscles of the back of the neck. There was slight headache, no impairment of intellect, but noted disturbance of vision.

The patient on endeavoring to read was able to see every line on the paper except the one he was trying to read. And seeing a man approaching him on the street he could easily discern every part of his body except his head. His sexual propensities were unduly excited, and for several hours he was greatly annoyed with constant erections. The patient complained of a saline taste in the mouth. There were no symptoms of any gastric irritation—no diarrhœa, polyuria or peculiar cutaneous eruption. There was excessive appetite, and the urine was increased in quantity and of high color. These symptoms, which lasted for nearly six hours, gradually subsided with no other treatment save a small dose of morphine to relieve the intense pain.

The patient having entirely recovered, was again put upon ten grains of iodide of potassium three times a day, but with no such results as before.

In a discussion of iodic salivation at the College of Physicians of Philadelphia, a number of cases were referred to in which salivation had followed the use of iodide of potassium, but in most of these cases the patients had been previously salivated with mercury. This patient has never suffered from mercurial salivation, and this case is undoubtedly one of pure iodic salivation.

Dr. McGaughey reports a case of a woman (*Phila. Med. Times*), twenty-four years old, in whom a dose of ten grains of iodide of potassium produced dry throat, loss of articulation, swelling of the neck, tremendous salivation and delirium, which passed off in the course of four or five hours. I know of no other cases except this, and the one I have just reported, where so small a dose of iodide of potassium has caused such intense salivation.

